# Prevalence and factors associated with substance use among HIV positive youth attending HIV care and treatment centers in Dodoma, Tanzania

**DOI:** 10.1186/s12981-022-00485-w

**Published:** 2022-12-24

**Authors:** Zahra Morawej, Azan Nyundo, Ally Kinyaga, Veneranda Kirway, Sophia Kagoye, Andrew Turiho, Noeline Nakasujja

**Affiliations:** 1 Department of Psychiatry, Hubert Kairuki Memorial University, Dar Es Salaam, Tanzania; 2 Department of Psychiatry and Mental Health, School of Medicine and Dentistry, The University of Dodoma, Dodoma, Tanzania; 3 Department of Surveillance, Monitoring and Evaluation, Centre for Reforms, Innovation, Health Policies and Implementation Research (CeRIHI), Dodoma, Tanzania; 4 PRAXIS for Health and Development, Dar Es Salaam, Tanzania; 5 National Institute for Medical Research, Mwanza, Tanzania; 6 Department of Psychiatry, Makerere College of Health Sciences, Kampala, Uganda

**Keywords:** Substance use, HIV, HIV positive youth, Prevalence, Tanzania

## Abstract

**Background:**

Substance use among people living with HIV is associated with poor health, social, and psychological outcomes. This study assessed the prevalence of substance use and associated factors among youth attending HIV care and treatment centers (CTCs) in Dodoma, Tanzania.

**Methods:**

This cross-sectional study was carried out in Dodoma, Tanzania, from February to April 2020 among youth aged 15–24 attending HIV CTCs. Data was collected using sociodemographic, WHO ASSIST Version 3.0, BDI II, and SERAD questionnaires. Data analysis was done using Stata 17. Descriptive statistics were used to summarize continuous and categorical variables. Univariable and multivariable logistic regression analyses were conducted to determine factors independently associated with substance use.

**Results:**

The prevalence of substance use was relatively low (6.6%). Older youth (20 to 24 years) were 2% less likely to use substances compared to the younger ones (15 to 19 years) (AOR: 0.07; 95% CI 0.01, 0.83). There were statistically significant decreasing odds of substance use with every year increase in age at HIV diagnosis (OR: 1.66; 95% CI 1.14, 2.41). Being unemployed was statistically significantly associated with decreased odds of substance use among this population (OR: 0.03; 95% CI 0.02, 0.33). Youth who had detectable viral loads were significantly more likely to use substances compared to those with undetectable viral loads (AOR: 12.9; 95% CI 1.07, 156.05).

**Conclusions:**

Despite the low prevalence of substance use found in this study, it is important to note that late age of HIV diagnosis, employment, and detectable viral load negatively impacted HIV positive youth with regards to substance use. It is recommended that CTCs emphasize routine screening for substance use among youth who have detectable viral loads.

## Background

HIV infection disproportionately affects young people, with individuals between the ages of 16 and 24 demonstrating the highest rates of new HIV infections compared to other age groups [1]. In 2020, approximately 1.75 million adolescents between the ages of 10 and 19 were living with HIV worldwide, with sub-Saharan Africa accounting for 88% of this population [2]. In 2020, 410,000 young people between the ages of 10 and 24 were newly infected with HIV, of whom 150,000 were adolescents between the ages of 10 and 19 [2]. Previous studies have also demonstrated a high prevalence of substance use among young people living with HIV [3–5].

Substance use has become one of the major growing public health and socioeconomic issues worldwide, particularly in developing countries [6, 7]. Substance use typically begins during adolescence, and patterns of behavior related to substance use are likely to be established during this time; those patterns can lead to numerous problem behaviors and health risks affecting the life course of these individuals [8, 9]. Structural neuroimaging studies suggest neural consequences may be associated with substance use [10, 11]. For example, smokers and cocaine users have exhibited lower gray matter volume than non-smokers and people who do not use cocaine [11–13]. Studies have also shown that heavy alcohol use reduces gray matter loss in different brain areas, including the frontal cortex and striatum [11, 14, 15]. Studies have also noted that adolescents and young adults who use substances during the adolescent phase of development show poor neuropsychological function on tests of inhibition and working memory [16–18]. This picture is further complicated by the presence of HIV infection among adolescents and young adults. Substance use among youth living with HIV (YLWH) has been linked to several negative health outcomes, including poor adherence to anti-retroviral therapy (ART), which can result in subsequent deterioration in health and the development of AIDS, culminating in mortality [19–23]. Substance use in this vulnerable population is also associated with the likelihood of engaging in risky sexual behaviors such as unprotected sexual intercourse, which increases the likelihood of developing new infections in HIV-negative youth [3, 24, 25]. Studies have shown that substance use among people living with HIV can hinder successful progress along the HIV care continuum, such as viral load suppression, the absence of opportunistic infections, improved quality of life and productive living, and the prevention of the development of AIDS [26–33].

A study conducted in the United States of America assessing the prevalence and correlates of substance use among youth (ages 12–26) living with HIV found that 32.9% used tobacco, 27.5% used marijuana, 21.3% used alcohol, and 22.5% reported any other illicit drug use [34]. A study in Uganda assessed HIV-positive adolescents aged 10–18 years for use of substances of abuse and found that 2.4% used alcohol and 2.4% used inhalants [35].

The majority of studies addressing the use of substances among YLWH have been carried out in high-income countries [34, 36]. Studies conducted in low- and middle-income countries have primarily focused on substance use among adults and not youth. Substance use studies among HIV-positive people conducted in Tanzania have primarily focused on injection drug use and not the full spectrum of both injectable and non-injectable substances, thereby leaving an information gap. Substance use among YLWH poses major public health concerns as it is associated with the presence of psychiatric disorders such as depression and anxiety and also presents a significant barrier to ART adherence, thereby hindering successful HIV disease prevention and management [37–39]. Despite substance use among YLWH presenting a public health concern, inadequate interventions have been directed towards this population, with most substance use interventions focusing on a purely psychiatric setting in adults [34]. Understanding the association between these factors will help plan successful and relevant HIV prevention and management interventions for this particular population. This study aimed to determine the prevalence and risk factors of substance use among HIV-positive youth attending care and treatment centers in Dodoma, Tanzania.

## Methods

### Study design and setting

This was an analytical cross-sectional study conducted across 4 care and treatment centers (CTCs) in Dodoma, the capital city of Tanzania, from February to April 2020. The prevalence of HIV in the Dodoma region is 2.9%, and that of substance use among youth (regardless of HIV status) is 14.6% [40, 41]. The participating CTCs were purposefully selected: Makole Health Center, Dodoma General Hospital, Mirembe Hospital, and St. Gemma. All centers except Dodoma Regional Referral Hospital review adolescents and youth (ages 10–24) once a month on the last Saturday of the month. Dodoma General Hospital conducts clinics for adolescents and youth living with HIV twice a month. The centers cater to different numbers of clients, the largest being Makole Health Center. All centers provide basic HIV services such as dispensing of ART, testing CD4 and viral load counts, and providing health education. Clients also receive mental health services such as psychoeducation and counseling (individual and group) on adherence, substance use, and risky sexual behaviors. Other services provided by the clinics involve activities and games, refreshments, and clubs based on the different interests of the clients.

### Participants

We enrolled youth ages 15–24 who were confirmed to be HIV positive and attending selected HIV care and treatment centers in Dodoma. They were aware of their HIV status and were able to give consent (18–24 years), as were emancipated minors (< 18 years). Youth aged 15–17 years gave their assent with the consent of their guardians. In instances where the guardians were unable to present to the clinic to give assent, mostly citing transportation challenges, the youth was excluded from participation in the study. Using the Leslie Kish formula for cross-sectional studies, a total sample of 211 participants was recruited from the 4 centers using a probability proportionate to size sampling technique per center followed by a systematic sampling method for each sampling frame [42].

### Measures

Sociodemographic variables comprised of age (completed years), sex (female or male), race/tribe (Gogo or other), religion (Muslim or Christian), education level (no formal education, primary level secondary or above), occupation status (unemployed, student or employed), orphan status (both parents alive or one or both parents died), and marital status (single, married, separated/divorced, or widowed). Information on clinical variables was obtained from patients’ CTC records; information was collected about the most recent viral load (within the last 6 months), most recent CD4 count (within the last 6 months), World Health Organization (WHO) HIV clinical staging, the ART regimen that they were currently on, the duration of ART use, and their age at diagnosis of HIV. Cut-offs for viral load and CD4 counts were adopted based on the Standard Treatment Guidelines and the National Essential Medicines List for Tanzania Mainland designates a viral load of < 40 copies/mL as undetected and ≥ 40 copies/mL as detected. The Standard Treatment Guidelines for Tanzania also categorize CD4 counts into two groups: < 200 cells/mm^3^ and ≥ 200 cells/mm^3^. This categorization helps guide clinicians in making decisions about issues such as treatment failure and HIV disease progression to AIDS and allows them to intervene accordingly.

Substance use was assessed using the Alcohol, Smoking, and Substance Involvement Screening Test (ASSIST). In this study, "substance use" referred to the non-medical use of any substance falling into categories of the ASSIST over the past 3 months prior to the study. These substances were: alcoholic beverages, tobacco products, cannabis, cocaine, amphetamine-type stimulants, inhalants, sedatives, hallucinogens, opioids, and others. The ASSIST is an 8–item questionnaire that assesses lifetime use of substances, substance use in the past 3 months, and injection drug use. The ASSIST also assesses the frequency of substance use. The ASSIST was developed by the World Health Organization and an international team of substance use researchers. It was designed for use by health care workers in a range of health care settings [43]. The ASSIST has shown a specificity range of 50–96% and a sensitivity range of 54–97% across the different substances it assesses [44]. In this study, any participant who scored 3 or above was considered a substance user.

Participants were assessed for depressive symptoms using Beck’s Depression Inventory, Second Edition (BDI-II). The BDI-II is designed for individuals aged 13 and above. It is a 21–item multiple-choice self-report instrument intended to assess the existence and severity of symptoms of depression as listed in the American Psychiatric Association’s Diagnostic and Statistical Manual of Mental Disorders, Fourth Edition [45]. The BDI-II has demonstrated high reliability regardless of its population. It has a high coefficient alpha (0.93), its construct validity has been established, and it is able to differentiate people with depression from those without [46].

Antiretroviral therapy (ART) adherence over the past month was assessed using the Self-Reported Adherence (SERAD) questionnaire. The SERAD is a self-reported instrument designed to provide easier adherence measurement. It is a 14-item tool that evaluates different time frames and possible reasons for medication non-adherence. The SERAD has been validated for use in the adult HIV-positive population [47]. The SERAD provides different possible factors for non-adherence to ART, and if a participant checks at least one of these factors, then they have not adhered to ART. Self-reported adherence to ART has been previously used in different clinical settings and has proven to be a valid method of adherence assessment among YLWH [48, 49].

### Statistical analysis

Data were entered using Epidata and analyzed using Stata 17 [50]. Measures of central tendency with respective measures of dispersion were used to summarize continuous variables like age, ART duration, CD4 count, and viral load. Frequency and percentages were used to summarize categorical variables. A chi-square (for > 5 expected observations) or Fisher’s exact (for < 5 expected observations) was used to evaluate an independent association between the categorical exposures and the main outcome of interest (substance use). We employed an adjusted logistic regression in the crude and adjusted analyses to determine factors associated with substance use. Independent variables with a *p*-value < 0.25 and priori confounders from the crude analysis were entered into the multivariable model to determine independent factors associated with substance use (*p*-value < 0.05). Confounders that were controlled in the multivariable model included marital status, occupation, education level, age at diagnosis of HIV, duration on ART, and viral load [5, 34]. All variables except the BDI score were analyzed as categorical variables (the BDI score was analyzed as a continuous variable). The CD4 and WHO HIV clinical staging variables were not included in the multivariable model because they resulted in quasi-complete separation of other variables. We opted to remove CD4 count and use the viral load instead, which is a more reliable predictor of clinical outcomes for people living with HIV [51–53].

## Results

### Sociodemographic characteristics of study participants

The mean age of participants was 19 years (SD = 2.5); a majority (63.5%) were aged 15 to 19 years. More than half (59%) were female, and a majority (66.8%) had secondary education or above (Table 1).

**Table 1:** Sociodemographic characteristics of study participants (N = 211)

Characteristic	Frequency	Percentage
Age		
15–19	134	63.5
20–24	77	36.5
Mean = 19 years (SD = 2.5)		
Sex		
Male	87	41.2
Female	124	58.8
Marital Status		
Single	198	93.8
Married/Sep/Widowed	13	6.2
Occupation		
Employed	54	25.6
Unemployed	68	32.2
Student	89	42.2
Education level		
Non formal/primary	70	33.2
Secondary or above	141	66.8
Religion		
Muslim	69	32.7
Christian	142	67.3
Tribe		
Gogo	76	36
Other	135	64
Orphan status		
Both parents alive	72	34.1
One/both parents died	139	65.9

### Clinical characteristics of study participants

About 6.6% of the youth reported substance use during the past 3 months, with alcoholic beverages comprising the majority of substance use (57%). The median depressive symptom score was 1 (IQR = 0,4). The median age at diagnosis of HIV was 11 years (IQR = 9,12) with the majority of the study participants (81.2%) being diagnosed with HIV below the age of 10 years (Table 2).

**Table 2:** Clinical Characteristics of study participants (N = 211)

Characteristic	Frequency	Percentage
Substance use in the past 3 months (n = 204)		
No	190	93.4
Yes	14	6.6
Type of substance used (n = 14)		
Tobacco	6	42.9
Alcoholic beverage	8	57.1
Frequency of substance use (n = 14)		
Once/twice	10	71.4
Monthly	4	28.6
BDI II Score^a^		
Median = 1 (IQR: 0,4)		
Age at diagnosis of HIV (n = 202)		
< 10 years	164	81.2
≥ 10 years	38	18.8
Median = 11 years (IQR: 9,12)		
WHO HIV clinical stage		
Stage I	201	95.3
Stage II & III	10	4.7
ART regimen		
1st line	208	98.6
2nd line	3	1.4
Duration on ART		
Between 1 & 6 months	6	2.8
> 6 months	205	97.2
CD4 count		
< 200 cells/mm3	14	6.6
≥ 200 cells/mm3	197	93.4
Mean = 559.5 (SD = 226.0)		
Viral load		
Undetected	197	93.4
Detected	14	6.6
ART adherence		
Adherent	132	62.6
Non-adherent	79	37.4
ART non-adherence factors (n = 79)		
Patient factors	76	96.2
Other factors	3	3.8

^a^Analyzed as a continuous variable

### Prevalence of substance use by sociodemographic & clinical characteristics

Employed youths had a significantly higher prevalence of substance use (14.8%) compared to unemployed youth (3.8%). Youth that were diagnosed with HIV before the age of 10 had a significantly lower prevalence of substance use (1.2%) compared to those diagnosed at ages above 10 (28.9%). The prevalence of substance use was also significantly higher among youths with a CD4 count of less than 200 cells/mm^3^ and among those with detectable viral loads at 35.7% and 57.1%, respectively (Table 3).

**Table 3:** Prevalence of Substance use by Sociodemographic & Clinical Characteristics (N = 211)

Characteristic	Substance use (Yes) n (%) n = 14	Substance use (No) n (%) n = 197	*p*-value
Age			
15–19	8 (6.0)	126 (94.0)	0.609
20–24	6 (7.8)	71 (92.2)	
Sex			
Male	6 (6.9)	81 (93.1)	0.898
Female	8 (6.5)	116 (93.5)	
Marital status			
Single	12 (6.1)	186 (93.9)	0.209
Married/sep/widowed	2 (15.4)	11 (84.6)	
Occupation			
Employed	8 (14.8)	46 (85.2)	0.005
Unemployed/student	6 (3.8)	151 (96.2)	
Education level			
Non formal/primary	2 (2.9)	68 (97.1)	0.150
Secondary or above	12 (8.5)	129 (91.5)	
Religion			
Muslim	4 (5.8)	65 (94.2)	1.000
Christian	10 (7.0)	132 (93.0)	
Tribe			
Gogo	3 (4.0)	73 (96.0)	0.388
Others	11 (8.2)	124 (91.8)	
Orphan status			
Both parents alive	5 (6.9)	67 (93.1)	1.000
One/both parents died	9 (6.5)	130 (93.5)	
Age at diagnosis of HIV (n = 202)			
< 10 years	2 (1.2)	162 (98.8)	< 0.001
≥ 10 years	11 (28.9)	27 (71.1)	
WHO HIV stage			
Stage I	9 (4.5)	192 (95.5)	< 0.001
Stage II & III	5 (50.0)	5 (50.0)	
ART regimen			
1st line	13 (6.3)	195 (93.7)	0.187
2nd line	1 (33.3)	2 (66.7)	
CD4 count			
< 200 cells/mm^3^	5 (35.7)	9 (64.3)	0.001
≥ 200 cells/mm3	9 (4.6)	188 (95.4)	
Duration on ART			
Between 1 & 6 months	5 (83.3)	1 (16.7)	< 0.001
> 6 months	9 (4.4)	196 (95.6)	
Viral load			
Undetected	6 (3.1)	191 (96.9)	< 0.001
Detected	8 (57.1)	6 (42.9)	
ART adherence			
Adherent	7 (5.3)	125 (94.7)	0.315
Non-adherent	7 (8.9)	72 (91.1)	
ART non-adherence factors (n = 79)			
Patient factors	7 (9.2)	69 (90.8)	1.000
Other factors	0 (0.0)	3 (100.0)	

### Factors associated with substance use

In the crude analysis, youth characteristics including current age, occupation, age at diagnosis of HIV, WHO clinical staging, duration on ART, CD4 count, and viral load were significantly associated with substance use. In the adjusted analysis, however, fewer characteristics—current age, occupation, age at diagnosis of HIV, and viral load were significantly associated with substance use. Older youth (20–24 years) were less likely to use substances compared to the younger ones (15–19 years) (AOR: 0.07; 95% CI 0.01, 0.83). Unemployed youth were less likely to use substances compared to their employed counterparts (AOR: 0.03; 95% CI 0.02, 0.33). With regard to the age at diagnosis of HIV, the odds of substance use increased by 1.7 times (AOR: 1.66; 95% CI 1.14, 2.41) for youth diagnosed with HIV after the age of 10. It was observed that youth who had detected copies/mm^3^ of viral load were significantly more likely to use substances compared to those whose copies/mm^3^ of viral load were undetected (AOR: 12.90; 95% CI 1.07, 156.05**) (**Table 4**).**

**Table 4:** Factors associated with Substance use (Crude Analysis & Adjusted Analysis)

Characteristic	COR^a^ (95% CI)	*p*-value	AOR^a^ (95% CI)	*p*-value
Age				
15–19	1	0.610	1	0.035
20–24	1.33 (0.44, 3.99)		0.07 (0.01, 0.83)	
Sex				
Male	1	0.898		
Female	0.93 (0.31, 2.79)			
Marital status				
Single	1	0.009	1	
Married/sep/widowed	2.82 (0.56, 14.18)		0.03 (0.02, 0.33)	0.005
Occupation				
Employed	1	0.009	1	0.005
Unemployed/ student	0.23 (0.08, 0.69)		0.03 (0.02, 0.33)	
Education level				
No formal/ primary education	0.32 (0.10,1.45)	0.139	0.15 (0.01, 1.78)	0.132
Secondary or above	1		1	
Religion				
Muslim	1			
Christian	1.23 (0.37,4.07)	0.734		
Tribe				
Others	1	0.897		
Gogo	0.46 (0.13, 1.72)			
BDI II Score	1.04 (0.90, 1.21)	0.585		
Age at diagnosis of HIV	1.76 (1.39, 2.22)	<0.001	1.66 (1.14, 2.41)	0.008
WHO HIV Stage				
Stage I	1			
Stage II & III	21.33 (5.22, 87.24)	0.109		
ART regimen				
1st line	1	<0.001	1	
2nd line	7.5 (0.64, 88.25)		0.60 (0.01, 30.30)	0.796
Duration on ART				
Between 1 & 6 months	1		1	
>6 months	0.01 (0.00, 0.09)	<0.001	0.60 (0.01, 30.30)	0.796
Viral load				
Undetected	1		1	
Detected	42.44 (11.17, 161.20)	<0.001	12.90 (1.07,156.05)	0.044
ART adherence				
Adherent	1			
Non-adherent	1.74 (0.59, 5.15)	0.320		

COR-Crude Odds Ratio; AOR-Adjusted Odds Ratio

## Discussion

This study found a prevalence of 6.6% for substance use among HIV positive youth attending CTC. This prevalence is similar to a study done in Dar es Salaam among youth in the general population, which reported a prevalence of 7% [54]. The prevalence is also similar to a study done at HIV clinics which reported a prevalence of 5.6% [55]. One possible reason for this relatively low proportion of substance use among this population group is that they receive counseling at the CTCs. The counseling involves education on nutrition, exercise, the importance of adherence to medication, and avoiding substance use. Studies have shown that counseling on health and general well-being has a positive effect on decreasing substance use [56–58]. Given that discussing one’s substance use may be viewed negatively, the prevalence in this study may also be low due to social desirability bias. The most frequently used substance was alcohol, followed by tobacco. Other studies in different clinical settings have also reported alcohol as the most frequently used substance among HIV positive youth [55]. The youth in this study reported never having used cannabis. This is in contrast with other studies in high-income countries, which have reported cannabis as one of the most commonly used substances among HIV positive youth [34, 59]. Studies looking at HIV and substance use have noted the association between intravenous (IV) drug use and HIV [60–62]. This study did not report IV drug use among HIV positive youth attending the CTCs. This may be because Dodoma is located in the center of the country, making it difficult to serve as a point of import for opiates and other IV drugs as seen in the coastal region of Tanzania, where youth in Dar es Salaam and Zanzibar are more likely to use IV drugs [63–65].

Unemployed youth and students were observed to have significantly lower odds of substance use compared to employed youths. This is similar to other studies that have been conducted among YLWH in clinical settings [24, 34]. A possible explanation for this result is that school offers a protective environment against substance use and other risky behaviors, and thus, the majority of participants, being students, are protected from substance use. Studies have shown that employment is associated with higher rates of substance use among youth [66–68]. This may be because employed youth are not offered the same protection from parents, guardians, and schools as their student counterparts. Unemployment has been associated with low use of substance use among youth because these youth are unable to afford the cost of substances; it serves as a protective factor against substance use [34]. Interestingly, our study also found that for every year increase in age at diagnosis of HIV from birth, the odds of substance use decreased significantly. A possible explanation for this may be that the majority of youth in this sample were diagnosed with HIV before age 10 and had been attending CTC and were on ART for a long period of time, thus receiving education on substance use and HIV. Studies have shown that receiving education on substance use is more likely to result in decreased substance use [57, 58]. Our study further found that the odds of substance use were significantly higher among YLWH who had detectable viral loads. Some studies have noted that detectable viral loads are associated with more substance use and have suggested that late entry into HIV care and poor ART adherence may serve as potential intermediate factors between the two [34, 69–71].

To the best of our knowledge, this study is one of the first to address both injectable and non-injectable drug use among YLWH. Our study is not without limitations. Due to the cross-sectional nature of this study, temporal relationships between HIV infection and substance use could not be established. Some of the study instruments used, such as the BDI II and the SERAD, for the assessment of depressive symptoms and adherence to ART, respectively, have not been validated in the Tanzanian setting. Nonetheless, previous studies conducted in Tanzania have used the BDI II to assess for depressive symptoms among PLWH [72, 73]. The SERAD has not been used to assess compliance with ARTs among PLWH in Tanzania, but it has been specifically designed for the HIV-positive population [47]. Another possible limitation of this study is recall bias caused by inaccuracies in reporting past events by the study participants. However, we tried to mitigate recall bias by accessing the clinical information from participants’ records. We also asked participants to be as truthful as possible to minimize social desirability bias.

## Conclusions

The prevalence of substance use among HIV-positive youth attending CTCs in Dodoma was 6.6%. The decreasing odds of substance use with every year increase in age at HIV diagnosis and among employed youth were observed to be statistically significant. This study highlights important aspects of substance use among youth living with HIV. We recommend routine screening for substance use among youth attending CTCs and providing continued psychoeducation and counseling to enable timely detection of the behavior and promote abstinence from substance use. Concerned health care providers should engage with policymakers to enact and promote policies against substance use as the vice plays a role in late presentation to clinical settings, particularly for YLWH.

Clients at CTC who consistently present with low CD4 counts, detectable viral loads, higher WHO HIV clinical staging, and suboptimal adherence to ART should be monitored closely for the possibility of the use of substances. We also recommend that further studies be done to explore other psychosocial aspects associated with substance use among HIV-positive youth, such as mode of transmission, adverse childhood experiences, and caregiver/family dynamics.

## Data Availability

The datasets used and/or analyzed during the current study are available from the corresponding author on reasonable request.

## Abbreviations

ART: Antiretroviral therapy
ASSIST: Alcohol, Smoking and Substance Involvement Screening Test
BDI II: Beck’s depression inventory II
CTC: Care and treatment centers
HIV: Human immunodeficiency virus
IV: Intravenous
SERAD: Self-reported adherence
WHO: World Health Organization
YLWH: Youth living with HIV
